# Spinal cord homogenates from SOD1 familial amyotrophic lateral sclerosis induce SOD1 aggregation in living cells

**DOI:** 10.1371/journal.pone.0184384

**Published:** 2017-09-06

**Authors:** Edward Pokrishevsky, Ran Ha Hong, Ian R. Mackenzie, Neil R. Cashman

**Affiliations:** 1 Djavad Mowafaghian Centre for Brain Health, University of British Columbia, Vancouver, British Columbia, Canada; 2 Department of Pathology and Laboratory Medicine, University of British Columbia, Vancouver, British Columbia, Canada; Rutgers University, UNITED STATES

## Abstract

Mutant Cu/Zn superoxide dismutase (SOD1) can confer its misfolding on wild-type SOD1 in living cells; the propagation of misfolding can also be transmitted between cells *in vitro*. Recent studies identified fluorescently-tagged SOD1^G85R^ as a promiscuous substrate that is highly prone to aggregate by a variety of templates, *in vitro* and *in vivo*. Here, we utilized several SOD1-GFP reporter proteins with G37R, G85R, or G93A mutations in SOD1. We observed that human spinal cord homogenates prepared from SOD1 familial ALS (FALS) can induce significantly more intracellular reporter protein aggregation than spinal cord homogenates from sporadic ALS, Alzheimer’s disease, multiple system atrophy or healthy control individuals. We also determined that the induction of reporter protein aggregation by SOD1-FALS tissue homogenates can be attenuated by incubating the cells with the SOD1 misfolding-specific antibody 3H1, or the small molecule 5-fluorouridine. Our study further implicates SOD1 as the seeding particle responsible for the spread of SOD1-FALS neurodegeneration from its initial onset site(s), and demonstrates two potential therapeutic strategies for SOD1-mediated disease. This work also comprises a medium-throughput cell-based platform of screening potential therapeutics to attenuate propagated aggregation of SOD1.

## Introduction

Over the past decade, an increasing number of *in-vivo* and *in-vitro* studies have identified prion-like mechanisms contributing to the spread of ALS pathogenesis from its initial focus/foci sites observed in disease [1–6]. For instance, aggregates composed of mutant SOD1 can penetrate into cells through macropinocytosis and nucleate aggregation of soluble cytoplasmic mutant SOD1 protein [7], and overexpression of mutant SOD1 protein in human cells can trigger the misfolding of endogenous wild-type SOD1 in the transfected cells [8]. Studies have also demonstrated that once SOD1 is triggered to misfold and/or aggregate inside cells, it can propagate intercellularly by hijacking the exosomal machinery or through macropinocytosis [1, 7]. Additionally, *in vivo* experimental transmission of SOD1-mediated motor neuron disease was first demonstrated in 2014, where intra-spinal injection of SOD1^G93A^ spinal cord homogenates into mice expressing SOD1^G85R^-YFP resulted in spinal motor neuron aggregation of SOD1^G85R^ -YFP and degeneration [3]. Most recently, the same group demonstrated the formation of fluorescent aggregates in organotypic spinal cord slice cultures prepared from SOD1^G85R^-YFP mice when incubated with spinal cord homogenates from SOD1-A4V patients, but not sporadic ALS (SALS) [9].

Here, we used spinal cord homogenates prepared from a total of four SOD1-FALS (A4V, D90A, G93S, I113T), three SALS, one healthy control, and three non-ALS controls (AD, MSA), to show that only homogenates prepared from SOD1-FALS can effectively trigger the aggregation of chimeric SOD1-GFP protein with G37R, G85R or G93A mutations in the SOD1 moiety. We also found that the SOD1 misfolding-specific monoclonal antibody 3H1, and the small molecule 5-fluorouridine, can attenuate the induction of SOD1-GFP aggregation by SOD1-FALS homogenates.

## Methods and materials

### Cell culture

Human embryonic kidney cells (HEK293FT; ATCC, Manassas, VA) were cultured in complete Dulbecco’s Modified Eagle Medium (DMEM) containing 10% FBS, 100 U/mL penicillin, 100 μg/mL streptomycin and 2 mM L-glutamine (ThermoFisher Scientific, MA, USA). For immunofluorescence studies, cells were grown in 24 well plates with cover slips or in black 96 well plates with glass bottom. To test the potency of the various tissue homogenates to seed aggregation of the SOD1-GFP protein in living cells (plasmids were a gift from Elizabeth Fisher [10]), we transfected pre-plated HEK293FT cells with the chimeric reporter protein using Lipofectamine LTX (ThermoFisher Scientific, MA, USA), according to manufacturer’s instructions.

### Tissue preparation and incubation with living cells

Research involving human subjects was approved by the ethics review board of the University of British Columbia, and included written consent from participants. We performed tissue extraction on the following tissues: four SOD1-FALS (A4V, disease duration: 2 years; D90A, disease duration: 17 years; G93S, disease duration: 6 years; I113T, disease duration: >10 years), three SALS, two Alzheimer’s disease (AD), one Multiple System Atrophy (MSA), and one healthy control. We chose AD and MSA as negative controls as both of these disorders have been studied for their prion-like features [11, 12], and their neurodegenerative nature that presents overall stress conditions. Tissue homogenates were prepared by first cutting ~0.1g of flash frozen human spinal cord tissue (C- or T-spine) and adding it to 9-parts of cold PBS supplemented with protease inhibitors (Roche Diagnostics, IN, USA). Each tissue was then homogenized 3x for 20 sec with 40 sec breaks (on ice), and sonicated once for an additional 15 sec. Homogenized and sonicated tissue was spun down at 1,000 x g for 5 min and the supernatant was aliquoted into fresh tubes. Total protein concentration in each homogenate was determined using a standard BCA assay, and adjusted between the samples in order to ensure that equal amount of protein is later added to the cell cultures. Homogenized tissues were stored in -80°C and each aliquot was only used for one experiment. Homogenates were added drop-wise to the transfected cells 4–6 h post transfection. Prior to the addition, homogenates were mixed with the appropriate volume of Lipofectamine 2000 (ThermoFisher Scientific, MA, USA) in serum reduced media (ThermoFisher Scientific, MA, USA). The cells were then incubated for 48 h in a 37°C humidified incubator supplemented with 5% CO_2_. For experiments that tested the efficacy of 3H1 and 5-FUr, the compounds were added to the transfected cells at the indicated final concentration 30–45 min prior to the addition of the homogenates. In order to estimate the efficacy of 3H1 and 5-FUr as therapeutic molecules, we included an additional control (referred to as “background”), where induced aggregation of the reporter protein was monitored in the presence of transfection reagent alone (no homogenate). Due to the scarce amounts of A4V-SOD1 tissue, it was only included in the induction studies.

### Inclusion counting algorithm using microscopy

Images were acquired using an inverted AxioObserver microscope (Carl Zeiss AG, Germany) with a motorized stage, and exported for analysis as a high resolution JPG files. For these studies, we used the Zen Blue (Carl Zeiss AG, Germany) to prepare a 96 well template with four global focus points. The template was calibrated for every new plate, and each focal point was adjusted in order to determine the focus plane of the plate. Our focusing strategy, along with the Definite Focus (Carl Zeiss AG, Germany), ensured that each well is then in focus. In order to quantify induced aggregation of SOD1 using our reporter protein, we developed an ImageJ-based algorithm that reliably counts inclusions based on the area that inclusions occupy normalized to total expressed GFP (S1 Fig). Briefly, to count inclusions and their area, the algorithm first converts each image into 8-bit images, performs a local background subtraction, thresholds based on pre-set settings, and counts the number of inclusions (and their size) based on a predefined size. In order to normalize induced aggregation, a parallel algorithm estimates the total area occupied by reporter protein fluorescence with a low threshold. The percentage of aggregation is reported as a ratio of area of inclusions divided by total area of fluorescence.

### Immunohistochemistry

Immunohistochemistry was performed using a monoclonal primary antibody raised against misfolded SOD1 (DSE2-10E11C11, 1:500 dilution, following microwave pre-treatment) on 5 mm thick sections of formalin fixed, paraffin embedded tissue sections using the Ventana BenchMarkH XT automated staining system (Ventana, AZ, USA) and developed with aminoethylcarbizole (AEC) or diaminobenzidine (DAB).

### Statistical analysis

Statistical significance of induced aggregation of the reporter proteins in the presence of homogenates was established by first confirming the Gaussian distribution of the data, and then performing one-way ANOVA and Dunnett’s test for multiple comparisons. To test if the effects of 5-FUr and 3H1 were significant, we compared induced aggregation of the reporter protein in the presence or absence of the molecules using two-tail unpaired t-test (alpha used P<0.05).

## Results

**Fig 1:**
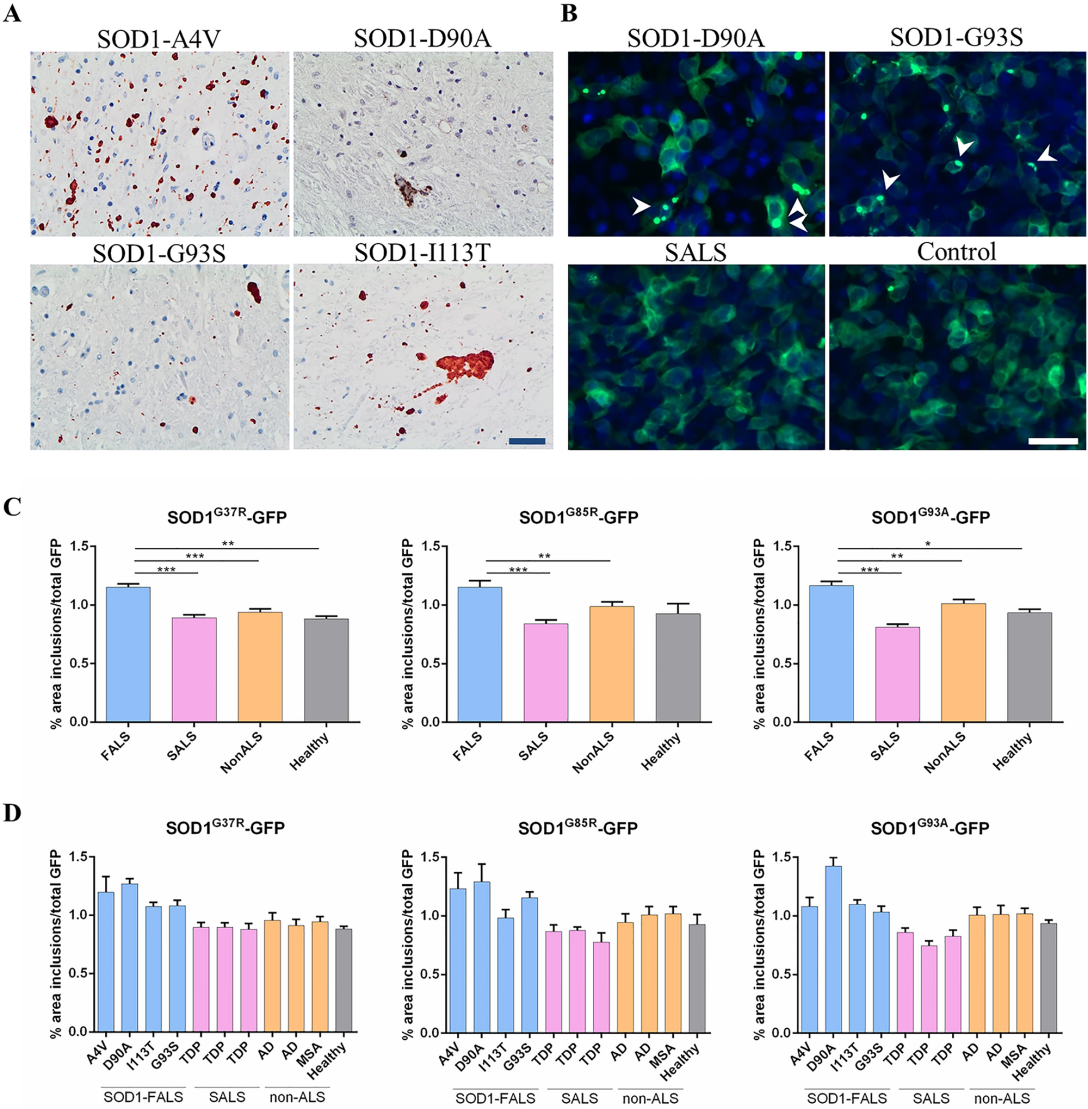
Homogenates prepared from familial ALS spinal cord tissue induce SOD1 aggregation. A) SOD1 inclusions in 4 SOD1-FALS cases (A4V, D90A, G93S, I113T) were confirmed by immunohistochemistry. B) Homogenates prepared from human spinal cord tissue were incubated with HEK293FT cells pre-transfected with the indicated reporter protein (G37R, G85R or G93A-based). Cells were imaged 48 h post treatment and analyzed for the presence of inclusions using our algorithm. Representative immunocytochemistry micrographs demonstrate induced aggregation of SOD1^G85R^-GFP in cells incubated with the indicated homogenate. Arrowheads point towards visible reporter protein inclusions. C) Summary of the effect of FALS, SALS and non-ALS control tissue homogenates on the reporter proteins. Bar graphs represent the percentage of reporter protein in inclusion form out of total reporter protein (area). Statistical significance was established using one way ANOVA followed by Dunnett’s test for multiple comparisons. D) Induced aggregation of the reporter protein using the individual homogenates grouped in (C). Each homogenate was tested 8–16 times with 2 technical repeat per run. *** p < 0.001, * p < 0.05. Scale bar: 40 μm.

We first used a monoclonal SOD1-misfolding specific antibody to confirm the presence of SOD1 aggregates in our SOD1-FALS cases (A4V, D90A, G93S, I113T) using immunohistochemistry (Fig 1A). Next, we prepared tissue homogenates from these four SOD1-FALS cases, three cases of SALS, as well as three non-ALS (AD, MSA) and healthy controls, and evaluated their ability to induce aggregation of SOD1-GFP reporter protein in living cells. We found that homogenates prepared from SOD1-FALS spinal cord tissues can trigger 30–40% more aggregation compared to homogenates from sporadic ALS patients, or 16–25% more compared to control samples, in all three G37R, G85R and G93A-based chimeric reporter proteins following 48 h incubations (Fig 1B and 1C). The quantification of inclusion formation was performed using a home-made algorithm where the area of reporter protein in inclusion form was normalized to total reporter protein expression (S1 Fig). Homogenates prepared from SALS spinal cord tissues did not induce significantly more reporter protein inclusion formation than non-ALS or healthy controls in this system. Interestingly, a close analysis of the effect of individual SOD1-FALS homogenates revealed variability in their ability to induce aggregation of SOD1-GFP reporter proteins; we found A4V and D90A spinal cord homogenates to be the most potent inducers in this system (Fig 1D).

**Fig 2:**
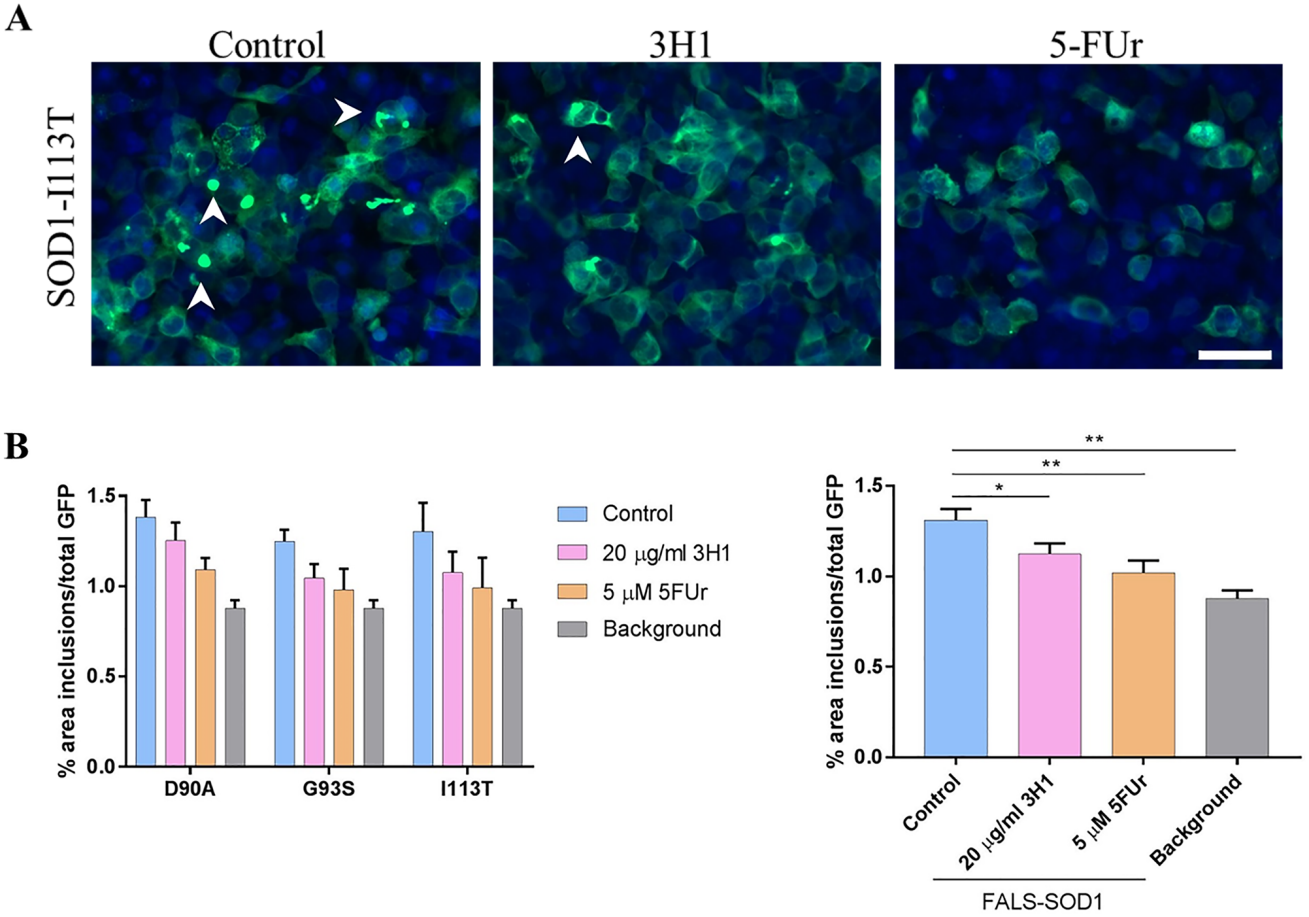
SOD1-misfolding specific antibodies and 5-fluorouridine reduce induced aggregation of SOD1-GFP by SOD1-FALS homogenates. Following a 4–6 h transfection of HEK293FT cells using SOD1^G85R^-GFP reporter protein, 5-FUr or 3H1 were added to the cells at a final concentration of 5 μM or 20 μg/ml, respectively, shorty prior to incubation with SOD1-FALS tissue homogenates. Cells were then incubated for an additional 48 h period, and analyzed for the presence of induced aggregates (**A**). We find that 5-FUr and 3H1 are effective at reducing induced reporter protein aggregation by SOD1-D90A, G93S or I113T spinal cord homogenates. The summary bar graph (FALS-SOD1) includes all the repeats from the SOD1-D90A, G93S or I113T. Unpaired t-test was used to demonstrate statistically significant reduction in detectable reporter protein inclusions between untreated and treated cells (**B**). Arrowheads point towards visible reporter protein inclusions. Five biological repeats were performed for each homogenate. *, p < 0.05; **, p < 0.01. Scale bar: 40 μm.

Previous studies using SOD1 misfolding-specific antibody 3H1 have demonstrated that it can be successfully used to block transmission of misfolded SOD1 in conditioned media collected from cells transfected with mutant SOD1 or TDP-43 [1, 2]. Moreover, co-crystallization studies showed that 5-fluouridine (5-FUr), an antimetabolite chemotherapeutic agent, can bind to SOD1 at the pocket surrounding its lone tryptophan [13], a residue that was linked to both template directed misfolding and toxicity of SOD1 [2, 8, 14]. In order to assess if induction of SOD1^G85R^-GFP aggregation in cells exposed to SOD1-FALS homogenates can be attenuated, we incubated the cells with 20 μg/ml of a 3H1, or with 5 μM 5-FUr [15], prior to adding the homogenates. Regardless of the particular SOD1 mutation, we found that both 3H1 and 5-FUr can significantly reduce the induced aggregation of SOD1^G85R^-GFP by SOD1-FALS homogenates (D90A, G93S, I113T), without affecting the expression levels of the reporter protein (Fig 2A). We estimate this reduction to be approximately 60% when background is subtracted prior to analysis (Fig 2B).

## Discussion

Based on the recently confirmed prion-like properties of SOD1 *in vitro* and *in vivo*, we sought to determine whether human spinal cord homogenates from SOD1-FALS or SALS could induce aggregation of SOD1^G37R^-GFP, SOD1^G85R^-GFP and SOD1^G93A^-GFP in living cells. We found that all three reporter proteins can be significantly aggregated in the presence of SOD1-FALS homogenates, with the exact level of induced aggregation dependent on the specific SOD1 mutation. Interestingly, little difference was observed in the induced aggregation of all three reporter proteins, suggesting that G85R is not the only permissive substrate for conversion. Consistent with recent findings that SOD1-A4V homogenate can trigger aggregation of SOD1^G85R^-YFP in organotypic spinal cord slice tissue [9], we find that SOD1-A4V can also trigger the aggregation of our reporter proteins in HEK293FT cell culture. Similar to the SOD1-A4V, a spinal cord homogenate prepared from SOD1-G93S triggered aggregation of all of the reporter proteins, consistent with the high propensity of the non-dimer interface mutant G93S to aggregate in patients [16]. Curiously, the SOD1-D90A homogenate appears to be the most potent inducer of reporter protein aggregation, despite the relatively slow disease progression (disease duration of 17 years), and the apparent small load of SOD1 aggregates in this patient. A recent study examined the effect of overexpressing the human SOD1-D90A mutation in mice, and found that SOD1-D90A can co-exist in two strains, with one strain forming more fragile and fragmentation-prone aggregates, as well as greater pathogenicity causing an earlier disease onset and faster progression [17]. It is possible that the human SOD1-D90A spinal cord homogenate used in this study was enriched in the aggressive strain, which would explain its rapid nucleation of the reporter proteins. In our assay, spinal cord homogenates from SOD1-I113T can also induce aggregation of the reporter protein; however the efficiency depends on the reporter protein employed. The third most common mutation in SOD1, I113T, is known to have an incomplete penetrance and is disruptive to SOD1 homodimerization on the molecular level [18, 19]. Our observation that SOD1^G37R^-GFP and SOD1^G93A^-GFP, but not SOD1^G85R^-GFP, are triggered to aggregate in the presence of this homogenate is consistent with the conformational selection mechanism previously proposed for prions [20]. In this model, conversion and aggregation is most efficient when the conformational ensemble of the input mutation (in this case SOD1-I113T) overlaps with the conformational ensemble of the reporter proteins (G37R and G93A). If this notion applies to SOD1 aggregation, it is intriguing to speculate that the input ensemble of SOD1-I113T possesses conformational states which are dissimilar to G85R, which is a natively misfolded monomer. Interestingly, we find that clinical duration of disease or the loads of misfolded SOD1 in the SOD1-FALS tissues do not directly correlate with the amount of induced reporter protein aggregation in cells exposed to the corresponding tissue homogenates. We propose several reasons for this lack of apparent correlation. First, regardless of its specific tissue source, mutant SOD1 seed in the homogenate may be sufficient to trigger seeding of the chimeric reporter protein, which then follows a patient-independent aggregation. Secondly, normally stable SOD1 aggregates in SOD1-FALS may break into the more volatile SOD1 oligomers during the extraction protocol. Recent studies have demonstrated that non-native SOD1 trimers are toxic to motor neuron-like cells, and that mutations that stabilize the trimeric form result in increased neuronal death [21, 22]. Lastly, mutant SOD1 in patients may form different aggregate strains with varying seeding properties [17, 23, 24], some of which are more favourably isolated using our extraction protocol.

Our results confirm the previous finding that spinal cord homogenates from SALS patients cannot efficiently induce aggregation of the reporter protein in living cells, despite the controversial presence of misfolded wild-type SOD1 in these tissues reported by some groups [8, 25, 26], but not others [27]. The only reporter protein that exhibited a minimal visual response to SALS homogenates was SOD1^G37R^-GFP, a destabilized mutation that has been previously suggested to aggregate as a different strain [3, 23]. This notion is further supported by the discovery that the epitope surrounding glycine at position 37 is a fibril forming segment, and that G37R substitution disables fibril formation [28]. There are potentially two distinct types of pathogenic SOD1: 1) aggregation-prone mutant SOD1 that is present in FALS-SOD1 patients. Previous studies showed that mutant SOD1 inclusions prepared *in vitro* can seed insoluble aggregation of cytosolic soluble mutant SOD1 [7], consistent with our current findings that mutant SOD1 seeds from FALS-SOD1 homogenates lead to aggregation of the reporter proteins; 2) misfolded wild-type SOD1 found in sporadic ALS without obvious ubiquitinated SOD1 inclusions. Induced misfolded wild-type SOD1 is present in cells overexpressing mutant SOD1 or TDP-43 as a non-aggregated soluble molecular species, and acquires the property of propagation between cells in a template directed fashion [1, 2]. We do not find this type to be an effective seed for aggregation in our system.

Binding of 5-FUr to SOD1 (at a pocket containing tryptophan-32), and attenuation of intermolecular SOD1 template directed misfolding using 3H1 antibody (generated against an unstructured electrostatic loop exposed only in misfolded SOD1) have been previously demonstrated [1, 2]; however, our current study is the first to demonstrate that both 5-FUr and 3H1 can successfully block seeding of SOD1 aggregation triggered by SOD1-FALS homogenates, despite the likely different targets and mechanisms of action. Given its plasma membrane permeability, we speculate that 5-FUr acts by binding to the seed in the tissue homogenates, as well as to the reporter protein substrate found inside cells, therefore preventing the homophilic intermolecular interaction mediated by tryptophan residues [8]. As for 3H1, its specificity for misfolded SOD1 and poor membrane penetrance suggest that it binds to mutant SOD1 in the tissue homogenates found in the extracellular environment. Finally, this work provides an innovative cell-based tool for screening potential drugs against induced SOD1 aggregation in SOD1-FALS patients.

## Supporting information

S1 Fig Description of our methodology for inclusion quantification. Fluorescence microscopy micrograph of HEK293FT cells 48 h after transfection with reporter protein and incubation with homogenate. A) Micrographs are first converted to 8-bit grey-scale images and local background is subtracted to emphasize inclusions and remove background fluorescence. Threshold is then set to identify inclusions (red in micrograph), followed by particle counting that identifies only thresholded inclusions of pre-determined size. B) Total reporter protein fluorescence is quantified using a similar process, but with lower threshold and more permissive size to capture entire cell fluorescence. Once all the parameters are adjusted based on representative images for each experiment, the rest of the micrographs are analyzed in a batch-form using the same set of optimized parameters.
(TIF)

S1 File Data underlying the findings described in this study. The dataset contains two sheets: 1. Data used to generate the graphs presented in Fig 1C and 1D; 2. Data used to generate the graphs presented in Fig 2B. The values represent the percentage of the area that is occupied by aggregated reporter protein out of total expressed reporter protein.
(XLSX)
