# Supplementary figures and images for: Spinal cord homogenates from SOD1 familial amyotrophic lateral sclerosis induce SOD1 aggregation in living cells
Source: PLoS One. 2017 Sep 6;12(9):e0184384. doi: 10.1371/journal.pone.0184384 (PMC5587256; doi:10.1371/journal.pone.0184384)

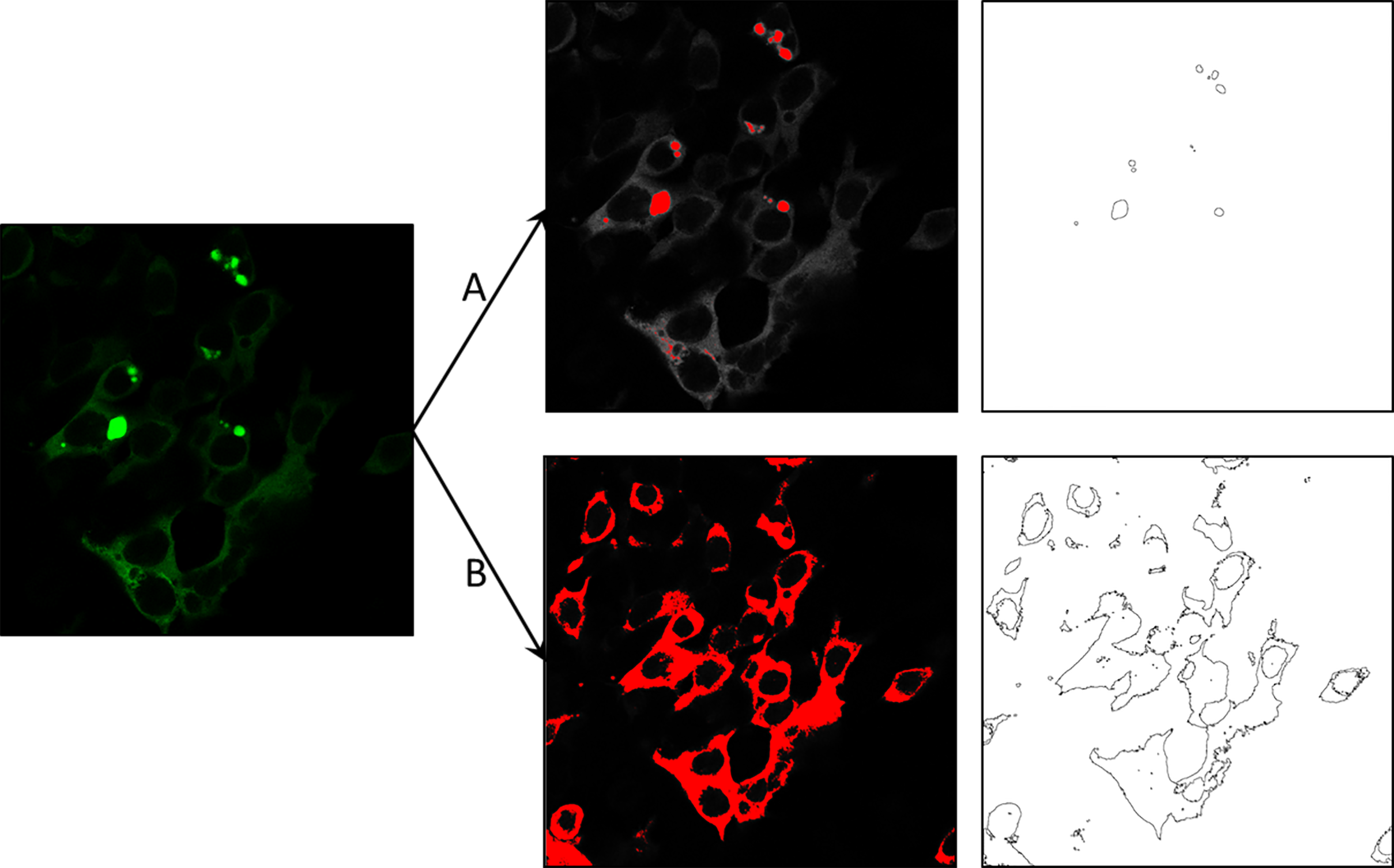

Supplement: S1 Fig — Fluorescence microscopy micrograph of HEK293FT cells 48 h after transfection with reporter protein and incubation with homogenate. A) Micrographs are first converted to 8-bit grey-scale images and local background is subtracted to emphasize inclusions and remove background fluorescence. Threshold is then set to identify inclusions (red in micrograph), followed by particle counting that identifies only thresholded inclusions of pre-determined size. B) Total reporter protein fluorescence is quantified using a similar process, but with lower threshold and more permissive size to capture entire cell fluorescence. Once all the parameters are adjusted based on representative images for each experiment, the rest of the micrographs are analyzed in a batch-form using the same set of optimized parameters. (TIF) [file pone.0184384.s001.tif]
